# Do response times add to self-reported craving? A secondary analysis of a neuromodulation trial

**DOI:** 10.3389/fpsyt.2026.1777646

**Published:** 2026-03-23

**Authors:** Larissa Vieira, Lorena Melo, Rodrigo Coelho Marques, Maisa Mendonça Silva, Paula Rejane Beserra Diniz

**Affiliations:** 1 Neuropsychiatry and Behavioral Sciences Postgraduate Program, Medical Sciences Center, Federal University of Pernambuco, Recife, Brazil; 2 Singular Psychiatry and Neuromodulation Clinic, Recife, Brazil; 3 SensoriMotor Laboratory, Jules-Gonin Eye Hospital, Fondation Asile des Aveugles, Department of Ophthalmology, University of Lausanne, Lausanne, Switzerland; 4 Department of Neuropsychiatry, Medical Sciences Center, Federal University of Pernambuco, Recife, PE, Brazil; 5 Department of Engineering Management, Technology and Geosciences Center, Federal University of Pernambuco, Recife, PE, Brazil; 6 Internal Medicine Department, Medical Sciences Center, Federal University of Pernambuco, Recife, PE, Brazil

**Keywords:** cue-induced craving, frontopolar cortex, implicit measure, medial prefrontal cortex, reaction time, symptom assessment, tobacco use disorder, transcranial magnetic stimulation

## Abstract

Craving is a core symptom and treatment target in tobacco use disorder. However, self-reported craving ratings often show weak or inconsistent associations with clinical outcomes, possibly because they overlook automatic motivational processes central to cue-reactivity. Response time to craving-item ratings has been proposed as an implicit marker of such processes, but its sensitivity to intervention and clinical relevance remain untested. In this exploratory secondary analysis of a randomized trial, tobacco-dependent adults completed an image-based cue-reactivity paradigm immediately before and after a single 1 Hz repetitive transcranial magnetic stimulation (rTMS) session targeting either the left frontopolar cortex (lFPC; n=12) or the vertex (Cz; n=11). We examined whether response time, modeled both as a mean-level measure and as a function of self-reported craving, was modulated by stimulation site and whether these indices were associated with smoking outcomes at baseline and one-week follow-up. Mean response time showed a significant Group × Time × Stimulus effect (χ²(1) = 8.64, p = 0.003): lFPC stimulation attenuated the typical post-exposure speeding observed in the vertex group, selectively for smoking cues. Models incorporating self-reported craving yielded convergent smoking-specific effects (Wald χ²(1) = 4.47, p = 0.034), but the curvature patterns were inconsistent and statistically fragile, limiting interpretability. Exploratory clinical analyses did not reveal reliable associations with smoking-related outcomes, although repeated nominal associations with tonic craving under smoking cues suggested a possible relationship. These findings provide initial evidence that response time may capture implicit components of cue-reactivity and function as a modulable behavioral endpoint sensitive to lFPC neuromodulation.

## 1 Introduction

Craving is broadly defined as the subjective experience of wanting to use a drug and represents the phenomenological expression of the motivational state that drives drug seeking (1, 2). As such, it stands as a core symptom and a central therapeutic target in substance use disorders (3, 4), playing, together with cue reactivity, a prominent role in relapse and drug-use behaviors (4–6).

Although neuromodulation approaches targeting craving-related circuits have shown efficacy for reducing cigarette consumption (7–10), meta analytic evidence indicates that they do not reliably reduce craving itself (10). Likewise, cue-reactivity studies frequently report weak or absent associations between self-reported craving and smoking outcomes (11–14), raising the hypothesis that these measures may fail to capture automatic motivational processes that contribute to relapse vulnerability (1, 2, 15–18).

Most cue reactivity studies rely on Likert-type or visual analogue craving ratings (13). Although considered the gold standard (1, 4, 5, 15), such self-reports depend on introspection and may overlook unconscious reactive components of the craving response (2, 16, 18). This limitation has motivated interest in complementary implicit measures that could index automatic components of cue reactivity and improve prediction of drug seeking and relapse (1, 4, 5, 17, 19).

Response time to craving-item ratings has emerged as one such promising candidate (16). Within attitude-strength and decision-making frameworks, response latency indexes representational accessibility and evaluative certainty, with faster responses occurring when internal states are more readily accessible in memory and decisional conflict is lower (20–25). An extensive theoretical and methodological literature addressing distributional properties, modeling strategies, and sources of cognitive and motor variance underscores the need for careful interpretation when applying latency measures to clinical constructs (26–29). Applied to craving, response time has been proposed as a complementary measure to self-reported craving that may capture aspects of drug-related motivational processing not fully reflected in explicit ratings (16).

In tobacco-dependent smokers, response time varies systematically with reported craving intensity, forming an inverted U shaped function in which slower latencies at moderate levels are interpreted as increased representational difficulty, reflecting reduced accessibility of internal craving states, evaluative conflict, or ambivalent motivational tendencies (16, 30). Moreover, dependent smokers show distinct latency patterns compared to less dependent individuals, and shorter latencies at equivalent levels of reported craving have been associated with higher nicotine dependence (16). However, whether response time is sensitive to biological interventions or provides incremental predictive value in tobacco use disorder remains unknown.

Repetitive magnetic transcranial stimulation (rTMS) over the left frontopolar cortex (lFPC) offers a compelling setting to test this possibility. The lFPC plays a central role in implicit motivational processing via valuation and salience attribution (31–35), is linked to symptom remission in addiction (36), and, in a previous randomized trial, a single 1-Hz session targeting this region reduced explicit craving exclusively for neutral cues in a cue-reactivity task when compared with vertex stimulation (37). Building on those findings, this incremental analysis examines whether response time offers additional leverage for characterizing implicit components of cue reactivity altered by neuromodulation.

The present work constitutes a secondary, theory-driven analysis of previously collected behavioral data (37). The original study focused on explicit craving outcomes. The current investigation, on the other hand, was further motivated by the conceptual possibility that latency measures might provide complementary information beyond explicit craving ratings within the same experimental framework. Therefore, in this exploratory study, response time was assessed both as a mean-level measure and as a function of subjective craving, testing its sensitivity to modulation of the lFPC relative to the vertex. We also examined the relationship between response time indices and smoking-related clinical measures at baseline and after one week. Our aim was to determine whether response time complements explicit craving ratings by indexing motivational processes that remain less accessible to introspection yet may hold relevance for intervention effects and relapse risk.

## 2 Materials and methods

### 2.1 Study design and data source

This secondary analysis used trial-level data from a previously published double-blind, randomized, parallel-group pilot clinical trial investigating the effects of a single 1-Hz rTMS session in tobacco-dependent adults (37). Participants had been randomly assigned to receive stimulation over the lFPC (Fp1; n = 12) or over the vertex (Cz, active control site; n = 11) (37–39). Before and immediately after rTMS, participants completed a cue reactivity task including smoking and neutral cues (37). For each trial, craving ratings (1–7, where 1 reflects minimal cue-elicited craving and 7 reflects maximal craving) and response times were recorded. Additional procedural details are described in the original report (37).

The sample comprised 23 participants who completed the original protocol. The initial trial was designed as a pilot investigation of short-term effects on self-reported craving and was not powered for the specific response-time analyses presented here.

### 2.2 Participants and clinical assessment

The target population consisted of right-handed adult smokers aged 18–70 years with more than 12 years of formal education to ensure adequate task comprehension. Participants were clinically evaluated by a psychiatrist prior to enrollment and met criteria for severe nicotine dependence, defined as a score > 6 on the Fagerström Test for Nicotine Dependence (FTND) and > 27 on the Questionnaire of Smoking Urges – Brief (QSU-B). Participants were excluded if they were currently using smoking cessation medications (bupropion, varenicline, or nortriptyline), presented other substance use disorders, reported neurological disorders, showed moderate or severe psychiatric comorbidities based on the Patient Health Questionnaire-9 (PHQ-9), the Generalized Anxiety Disorder-7 (GAD-7), and clinical interview, were pregnant or at risk of pregnancy, or had any contraindication to rTMS.

In addition, at baseline, participants also completed a cognitive status assessment (Montreal Cognitive Assessment; MoCA), and impulsivity was evaluated by the Barratt Impulsiveness Scale-11 (BIS-11). Years of smoking and self-reported cigarettes per day were also recorded. A seven-day follow-up reassessed FTND, QSU-B, and cigarette consumption for exploratory evaluation of short-term clinical change (37).

### 2.3 rTMS protocol

rTMS was administered according to the protocol of the original randomized clinical trial (37), and is summarized here to provide methodological clarity. rTMS was delivered using a MagPro R20 stimulator (MagVenture, Denmark) equipped with a figure-of-eight MCF-B70 coil. Resting motor threshold (RMT) was determined over the left primary motor cortex as the minimum intensity eliciting visible contralateral first dorsal interosseous muscle contraction in at least 5 out of 10 trials. Stimulation consisted of a single session of 1200 pulses delivered at 1 Hz. Intensity was set at 110% of RMT for the lFPC group and 90% of RMT for the vertex control group. Coil positioning followed the international 10–20 EEG system (Fp1 or Cz), with the coil placed tangentially to the scalp and the handle oriented posteriorly.

### 2.4 Data analysis

Analyses proceeded in four steps. All procedures followed established recommendations for reaction-time data and clustered designs (26, 27, 29, 40, 41). Full analysis scripts, model specifications, and diagnostic checks are provided in the **Supplementary Material**.

#### 2.4.1 Step 1 – Data preprocessing

Response times were first restricted to a physiologically plausible range (0.15–20 s) and then log-transformed to reduce skewness and stabilize variance (26, 27). To account for the temporal structure of the task, models included trial order within session and previous-trial latency as covariates, capturing practice/fatigue and short-range autocorrelation (26). Craving ratings were decomposed into a session-level mean and a trial-specific deviation using a Mundlak-type specification to separate between-session differences from within-person fluctuations (29).

Exploratory baseline visualizations aimed at reproducing previously reported latency–craving patterns (16) applied analysis-specific descriptive preprocessing and are reported separately in the **Supplementary Material**; all inferential models relied on the unified trial-level preprocessing framework described here, with additional trimming and threshold variations evaluated exclusively as robustness checks.

#### 2.4.2 Step 2 – Computing group differences in mean response time

Mean response time was examined with trial-level linear models including group, time (pre and post), and stimulus type (neutral and smoking cues), and their interactions. Mixed-effects models were initially attempted given the nested structure (26, 40), but proved inestimable. Final models, therefore, used ordinary least squares with participant-clustered robust standard errors (41). To quantify the Group × Time interaction in an interpretable metric, we summarized a Difference-in-Differences (DiD) ratio-of-ratios from the model-estimated marginal means. This index compares the proportional pre–post change in the lFPC group relative to the proportional change in the vertex group, providing a scale-free estimate of differential speeding.

#### 2.4.3 Step 3 – Measuring intervention effects on curvature

To test whether 1 Hz-rTMS modulated the shape of the latency–craving function, models included orthogonalized linear and quadratic components of trial-level craving deviation and their interactions with group and time (16, 26). Analyses were conducted separately for neutral and smoking cues. Curvature change was defined as the difference between post- and pre-stimulation quadratic coefficients. In occasional cases of local rank deficiency, an algebraically equivalent but simplified parametrization was used; curvature contrasts were invariant across specifications.

#### 2.4.4 Step 4 - Clinical association analyses

To evaluate clinical relevance and potential predictive value, two subject-level indices were derived for each stimulus category: geometric-mean latency, indexing typical response speed (26), and the individual quadratic coefficient, capturing nonlinear latency–craving dynamics as a candidate implicit marker of motivational processing. These indices were correlated with baseline craving, nicotine dependence, and daily cigarette consumption using non-parametric and partial correlations, each adjusted for a single confounder (age, years smoking, depressive/anxious symptoms, impulsivity, or cognitive status). This approach was intended to examine the robustness of associations to clinically plausible sources of confounding while maintaining model parsimony in a small sample. To control for multiplicity within this exploratory association layer, p-values were corrected within stimulus type using the Benjamini–Hochberg false discovery rate (FDR) procedure. Both nominal and FDR-adjusted p-values are reported.

Exploratory ANCOVA models then examined whether baseline or change in behavioral indices predicted clinical outcomes at Day 7, using HC3 standard errors, which mitigate small-sample bias in regression estimates (42). Given the pilot sample size, additional multivariable adjustment was not performed to avoid model overfitting. These predictive models were conducted in an exploratory framework.

All analyses were performed in Python 3 (Google Colab). Data handling relied on pandas and numpy, statistical modeling on stats models and patsy, and visualization on matplotlib. Additional diagnostics were conducted using scipy. The full executable analysis pipeline is available in the **Supplementary Material** (Script).

## 3 Results

### 3.1 Mean-level response time

A significant three-way interaction (Group × Time × Stimulus) emerged (χ²(1) = 8.643, p = 0.0033), indicating that pre–post changes in response time varied by group and stimulus type. For smoking cues, the vertex group showed a markedly larger reduction in response time (28%) compared to those in the lFPC group (6%), yielding a DiD ratio-of-ratio estimate of 1.30. In contrast, for neutral cues, both groups showed comparable reductions (lFPC: 24%; vertex: 16%; ratio-of-ratios = 0.90). A main effect of time was also observed across conditions (β = –0.172, p = 0.0069), reflecting a general speeding of responses from pre to post intervention.

### 3.2 Response time modeled as a function of craving

**Figure 1:**
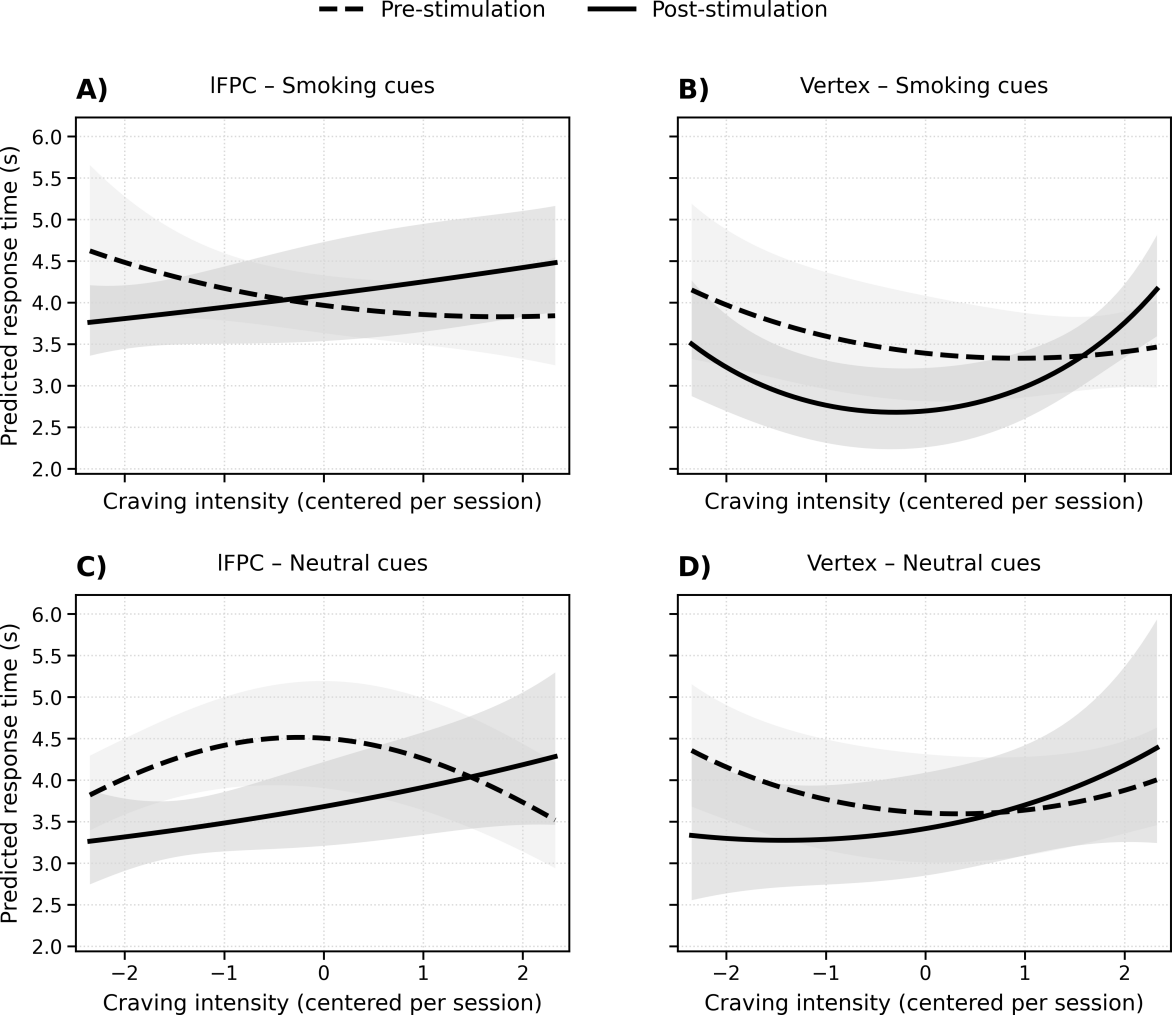
Predicted response-time functions by stimulation group and cue type. **(A)** (upper left) shows the lFPC group under smoking cues, and **(B)** (upper right) shows the vertex group under smoking cues. **(C)** (lower left) shows the lFPC group under neutral cues, and **(D)** (lower right) shows the vertex group under neutral cues. Curves represent model-based predictions from the trial-level analysis including linear and quadratic components of the session-centered craving intensity rating and adjustment for previous-trial response time. Dashed lines indicate pre-stimulation estimates; solid lines indicate post-stimulation estimates. Shaded areas indicate 95% confidence intervals. lFPC: left frontopolar cortex.

A significant group difference in curvature change emerged only for smoking-related cues (Wald χ²(1) = 4.47, p = 0.034), with the lFPC group showing post-stimulation flattening on the latency craving curve relative to vertex group. For neutral cues, no significant difference in curvature change was found between groups (Wald χ²(1) = 2.77, p = 0.096). Across all models, quadratic craving terms significantly improved overall model fit (joint quadratic block: Wald χ²(8) = 93.76, p < 0.001). However, the resulting curves were small in magnitude, heterogeneous across participants, and did not reliably form an inverted-U pattern (**Figure 1**; see **Supplementary Material** for diagnostic details and baseline tests contrasting linear and quadratic models).

### 3.3 Clinical associations

#### 3.3.1 Baseline correlations

Baseline correlations showed no associations with FTND or cigarettes/day (**Table 1**). Under smoking cues, curvature displayed the only significant bivariate effect, correlating with QSU-B (ρ = 0.47, p = 0.024). Geometric-mean latency also showed a trend in the same direction (ρ = 0.40, p = 0.061). No effects emerged under neutral cues.

**Table 1:** Baseline simple correlations with clinical variables.

Marker	Stimulus	Clinical variable	ρ	p
Response latency *(geometric mean)*	Neutral	Daily Cigarettes	0.216	0.323
		FTND	–0.136	0.537
		QSU-B	0.274	0.206
	Smoking	Daily cigarettes	0.238	0.275
		FTND	–0.135	0.538
		QSU-B	0.397	0.061
Curvature coefficient	Neutral	Daily cigarettes	–0.116	0.597
		FTND	–0.125	0.568
		QSU-B	0.153	0.486
	Smoking	Daily cigarettes	0.303	0.160
		FTND	–0.049	0.823
		QSU-B	0.470	0.024*

Simple Spearman correlations examining associations between baseline behavioral markers (response latency expressed as a geometric-mean measure and the curvature coefficient representing the quadratic term of the latency–craving function) and clinical indices of tobacco-use severity (QSU-B, FTND, and daily cigarettes) under neutral and smoking cues. All analyses used the full baseline sample (N = 23). QSU-B, Questionnaire of Smoking Urges – Brief; FTND, Fagerström Test for Nicotine Dependence; * Statistically significant at p < 0.05.

Across the 70 partial correlations performed (35 per marker, each adjusting for a single confounder), only seven yielded nominal significance before FDR correction, all involving QSU-B under smoking cues (**Table 2**). For latency, partial effects ranged from ρ = 0.42–0.44 (p < 0.05; p FDR = 0.226). For curvature, partial effects ranged from ρ = 0.45–0.46 (p < 0.04; p FDR = 0.149). No partial correlation with FTND or cigarettes/day approached significance, and no effects appeared under neutral cues.

**Table 2:** Partial correlations between behavioral markers and clinical variables.

Marker	Stimulus	Clinical variable	Confounder	ρ (partial)	*p*	*p*FDR
Response latency	Smoking	QSU-B	Years of smoking	0.425	0.043*	0.226
			GAD-7	0.416	0.048*	0.226
			BIS-11	0.436	0.038*	0.226
Curvature	Smoking	QSU-B	Age	0.461	0.027*	0.149
			Years of smoking	0.451	0.031*	0.149
			PHQ-9	0.446	0.033*	0.149
			GAD-7	0.459	0.028*	0.149

This table reports the partial Pearson correlations (ρ) that reached nominal significance (*p* < 0.05) before false-discovery-rate adjustment, each obtained by adjusting for a single confounder (age, years of smoking, depressive or anxiety symptoms, impulsivity, or cognitive performance). QSU-B, Questionnaire of Smoking Urges–Brief; GAD-7, Generalized Anxiety Disorder scale; PHQ-9, Patient Health Questionnaire–9; BIS-11, Barratt Impulsiveness Scale; FDR, false discovery rate; * Statistically significant at p < 0.05 (uncorrected).

#### 3.3.2 Predictive models

In the ANCOVA models predicting short-term clinical outcomes, many combinations could not be estimated because the models failed to converge or produced indeterminate parameters, typically due to singular covariance structures or insufficient variability within specific cells. Only models that yielded stable, estimable coefficients were retained for reporting (**Table 3**). Among these, the only nominal associations again involved QSU-B under smoking cues in the lFPC group: higher post-intervention curvature predicted lower follow-up QSU-B (β = –17.35, p = 0.049), and a similar trend emerged for the pre–post change in curvature (β = –13.82, p = 0.039). None of these effects survived FDR correction (p_FDR = 0.097 and 0.078, respectively), and no consistent patterns were observed for other outcomes or markers.

**Table 3:** Predictive ANCOVA models for short-term clinical outcomes yielding valid estimates.

Model	Outcome	β	SE	t	*p*	*p*FDR
Post-intervention curvature	QSU-B	-17.35	8.80	-1.97	0.049*	0.097
	FTND	0.13	1.14	0.11	0.910	0.910
	Daily cigarettes	0.21	0.24	0.86	0.390	0.780
Change (post–pre) in curvature	QSU-B	-13.82	6.69	-2.07	0.039*	0.078
	FTND	0.50	0.35	1.42	0.156	0.312
	Daily cigarettes	0.27	0.14	1.95	0.051	0.102

Predictive ANCOVA models examining whether post-intervention behavioral markers or their pre–post change predicted short-term clinical outcomes. All models were estimated within the *Smoking* condition and for curvature measured over the *lFPC* group. Only models yielding valid coefficient estimates are displayed (i.e., ≥10 valid trials per condition, non-singular HC3 covariance, and non-degenerate standard errors). Most models could not be estimated due to convergence failures or singular covariance structures and were therefore omitted. QSU-B, Questionnaire of Smoking Urges–Brief; FTND, Fagerström Test for Nicotine Dependence; lFPC, left frontopolar cortex; FDR, false discovery rate; * Statistically significant at p < 0.05 (uncorrected).

## 4 Discussion

### 4.1 Summary of main findings

This proof-of-concept intervention study provides preliminary evidence that response time to cue-induced craving ratings may serve as a modulable implicit marker of cue reactivity in tobacco dependence. After TMS, group differences emerged only for smoking cues, with the left frontopolar group showing less mean response-time speeding and a flatter response-time–craving curvature compared with the vertex group. This specificity to smoking cues contrasts with prior results in the same sample, in which frontopolar stimulation reduced explicit craving only for neutral cues (37). Together, these findings suggest that response time may capture implicit components of cue reactivity that are not reflected in explicit craving ratings, highlighting its potential as a mechanistic, intervention-sensitive behavioral index.

### 4.2 Psychometric considerations for interpreting response time as a behavioral endpoint

Interpreting these effects requires caution. In the broader psychometric literature, response time during Likert-type judgments is influenced by multiple sources, including properties of the evaluative process itself (e.g., representational strength or how closely one’s internal state matches the content being rated), item characteristics that affect temporal difficulty (e.g., syntactic complexity), and respondent-level tendencies that shape overall rating speed (28, 43–45). Although latency models carry systematic information, the effects of interest account for only a small portion of the variance (28). As a consequence, such patterns are fragile and typically require large samples and a balanced distribution of responses across the scale to emerge reliably (28). In contrast, it has been shown that response time during craving ratings can reflect implicit components of cue reactivity (16), providing conceptual support for treating response time as an intervention-sensitive endpoint. Rather than being treated solely as an ancillary psychometric correlate of item responding, response latency is framed in the present study as a potentially modulable behavioral endpoint that may shift when motivational or valuation processes are perturbed.

Within this framework, our results highlight response time itself, rather than the structure of the response time–craving function, as the more robust and interpretable endpoint. Although the inverted-U relationship between craving intensity and response time appeared in the collapsed baseline dataset, it was not stable when data were partitioned by group or modeled with subject-level covariates. The absence of a consistent non-linear structure reflects the known fragility of such psychometric effects and precluded a clear interpretation of how the intervention might have altered the internal relationship between craving intensity and response latency.

### 4.3 Mechanistic interpretation: lFPC modulation of implicit motivational processes

By contrast, the differential change in response time itself emerges as the clearest and most interpretable outcome. Whereas repeated exposure typically produces faster responding due to task familiarization (16), lPFC stimulation appeared to counteract this effect selectively for drug-related cues. Although the present data do not permit causal inference, this pattern invites a mechanistic hypothesis. Such a change may reflect greater deliberation or reduced automaticity when participants evaluated craving intensity for smoking cues after stimulation. One possibility is that frontopolar perturbation diminished the automatic motivational drive normally elicited by conditioned stimuli, thereby reducing the influence of subcortical incentive-salience signals and enhancing reliance on slower, more controlled evaluative processes.

This interpretation is consistent with the functional role of the left frontopolar cortex, which is preferentially engaged during the valuation of drug-associated stimuli and participates in processes such as incentive-salience attribution, goal-directed evaluation, and habit formation (8, 31, 32, 36, 46–54). Neuroimaging studies consistently show increased frontopolar and adjacent ventromedial prefrontal activation in response to drug cues and dynamic interactions with striatal and salience-network regions (8, 31, 55). Neuromodulatory studies also indicate that altering activity in this region can reduce craving, consumption, and cue-reactive neural responses (31, 35, 48, 55–57). Within this framework, the selective slowing of response time relative to task familiarization effects could reflect altered motivational-evaluative dynamics during craving appraisal. This possibility remains speculative and requires direct testing in larger, preregistered studies incorporating multimodal validation.

### 4.4 Distinguishing neuromodulatory effects from active-control artifacts

Despite these considerations, it is essential to establish that the differential slowing observed in the frontopolar group cannot be attributed to nonspecific effects of the active control site. Vertex stimulation is widely used as an adequate control for cognitive paradigms (38) and is not involved in valuation, salience attribution, or cue-induced motivational processing (6, 13, 38, 39, 58–63). Although vertex stimulation may occasionally reach adjacent pre-supplementary motor area (SMA) or SMA regions, which contribute to motor preparation and automatic action tendencies during cue reactivity (61, 64), such effects are typically small, diffuse, and non-selective, producing response time changes roughly tenfold smaller than those observed here (39). Moreover, SMA circuits do not compute value or incentive salience and would therefore be expected to influence response time uniformly, rather than generate the selective slowing restricted to smoking cues (59). The absence of group differences for neutral stimuli and the robust Group × Time interaction for smoking cues further indicate that vertex stimulation cannot account for the present findings. Instead, the pattern points to a frontopolar-specific attenuation of automatic motivational responding, rather than motoric or nonspecific TMS effects.

### 4.5 Methodological and conceptual contributions

Beyond mechanistic interpretations, the study also contributes methodologically by demonstrating that response time to self-reported craving is not only measurable at the trial level but also sensitive to intervention-induced change. Trial-level modeling and within-person decomposition of craving ratings (26, 27) maximized the information extracted from the available dataset and provided an analytical structure well-suited for small clinical samples.

The exploratory clinical analyses did not allow us to delineate a clear or statistically robust relationship between response-time indices and clinical outcomes. Although QSU-B showed recurring nominal associations under smoking cues across simple, partial, and predictive models, none of these effects survived correction for multiple comparisons. In the context of a small pilot sample and a multidimensional analytic framework, such recurrence may reflect correlated variability rather than a stable underlying association. Accordingly, these findings cannot be interpreted as evidence of clinical relevance, but they raise a question that warrants examination in larger, adequately powered studies.

Taken together, our results are consistent with the conceptual plausibility that the latency of explicit craving evaluations may represent a modulable component of cue reactivity, complementing explicit measures and extending prior work (16). Within the constraints of a pilot dataset, the most coherent pattern concerned mean-level latency modulation under left frontopolar stimulation. These findings support the conceptual plausibility of response latency as an intervention-sensitive dimension of cue reactivity, while leaving mechanistic clarification to future studies.

### 4.6 Limitations and future directions

The present findings should be interpreted as hypothesis-generating and proof-of-concept. The small sample size, the complexity of the analytic models, and the limited variability at extreme craving intensities constrained the precision of both mean-level and nonlinear response-time estimates, and contributed to the fragility of the exploratory clinical associations. Although vertex stimulation is unlikely to have influenced the results, its use as an active control still limits physiological specificity. Future studies should employ larger, adequately powered, and preregistered designs with placebo-controlled stimulation conditions and multimodal validation approaches (e.g. neurophysiological and psychophysiological measures) to establish specificity, mechanistic pathways, and clinical relevance. In particular, it remains to be determined whether response time can be considered as a reliable implicit endpoint in cue-reactivity research.

## 5 Conclusion

Response time to craving-item ratings appears to be sensitive to neuromodulatory effects, particularly as an independent mean-level measure, and may capture components of cue reactivity that remain less accessible through explicit self-report ratings. The selective modulation observed for smoking cues suggests that response time constitutes an experimentally manipulable endpoint, potentially indexing implicit motivational processes involving the left frontopolar function. Rather than serving as a clinical predictor at this stage, response time appears to reflect intervention sensitivity, positioning it as a potentially valuable conceptual and methodological addition to cue-reactivity research.

## Data Availability

The data analyzed in this study is subject to the following licenses/restrictions: The datasets analyzed in this study are not publicly available due to ethical and privacy restrictions involving human participants. The data were obtained from a previously conducted clinical study, and requests to access the data should be directed to the corresponding author and will be considered in accordance with the approved ethical protocol. The analysis code used in this study is provided as **Supplementary Material**. Requests to access these datasets should be directed to Larissa Vieira, larissapbvieira@gmail.com.
