# Supplementary material for: Do response times add to self-reported craving? A secondary analysis of a neuromodulation trial
Source: Front Psychiatry. 2026 Mar 23;17:1777646. doi: 10.3389/fpsyt.2026.1777646 (PMC13050832; doi:10.3389/fpsyt.2026.1777646)
Supplement: Supplementary file 2 [file DataSheet2.docx]

Supplementary Material

# Supplementary Methods

## Preprocessing of Response Time Data

Before conducting inferential modeling, we performed an initial diagnostic examination of the raw response-time data to ensure that the preprocessing steps were appropriate for latency measures. Following standard procedures in reaction-time research, we evaluated distributional characteristics within each experimental cell using Shapiro–Wilk tests, Levene variance tests, and non-parametric comparisons based on subject-level means. These exploratory checks (**Supplementary Figure 1** and Preprocessing Pipeline in **Supplementary Script**) were not part of the inferential analysis itself but served to guide the selection of transformations and covariates.

The raw response-time distributions showed the expected strong right skewness typical of human latency data. Log transformation substantially improved symmetry and reduced kurtosis (skewness from 2.05 to −0.60; kurtosis from 6.66 to 2.68), supporting its use as the primary modeling scale. This transformation is widely recommended for stabilizing variance and minimizing the undue influence of extreme values in reaction-time datasets (Baayen and Milin, 2010; Whelan, 2008).

Consistency checks also identified only three values out of 920 (0.33%) falling outside physiologically plausible limits (0.15–20 seconds). These trials were excluded from all trial-level analyses. Because their occurrence was negligible, no further trimming was warranted, consistent with the principle of minimal data loss.

To account for the intrinsic temporal structure of behavioral tasks, trial-level models incorporated two covariates that capture within-participant sequential dependencies: the position of each trial within the session, indexing practice- or fatigue-related effects, and the response time from the immediately preceding trial, which models short-range autocorrelation. These adjustments follow methodological recommendations for trial-level analyses of behavioral latencies and prevent serial dependence from biasing regression estimates (Baayen and Milin, 2010; Whelan, 2008).

For analyses examining the shape of the relationship between response time and self-reported craving intensity, craving ratings were decomposed into two components through a Mundlak-type adjustment: a session-level mean, reflecting stable differences between individuals and across pre- and post-intervention periods; and a trial-specific deviation from that mean, capturing moment-to-moment fluctuations (Mundlak, 1978). This separation enables the curvature parameter to reflect within-person reactivity rather than global differences in overall craving across sessions, which is particularly important when evaluating whether stimulation altered implicit aspects of cue reactivity (Laurenceau et al., 2025).

This unified preprocessing framework was applied to all trial-level inferential models. Analysis-specific preprocessing adjustments (e.g., stimulus-specific re-orthogonalization for curvature models, minimal exploratory trimming for Germeroth-style baseline visualizations, or participant-level aggregation for clinical correlations) are described in their respective sections to avoid redundancy.

## Exploratory Baseline Characterization

To assess whether our sample reproduced the inverted-U pattern previously described in tobacco users, we applied the same analytic procedure used to characterize the baseline association between response latency and momentary craving intensity (Germeroth et al., 2015). Only pre-intervention trials were considered in this step. Analyses were performed separately for neutral and smoking-related cues, first pooling all participants to obtain stable group-level patterns, and then repeating the procedure within each stimulation group to examine consistency of the functional shape.

For each stimulus category, scatterplots with locally estimated scatterplot smoothing (LOESS) were used to visualize the empirical relationship between craving intensity and response latency (**Supplementary Figure 2**). To formally test whether a quadratic specification provided a better approximation than a purely linear trend, we fitted polynomial regressions to participant-level averages for each craving level; corresponding model comparisons are reported in Supplementary Table 1.

This step served strictly as a descriptive confirmation that the baseline data exhibited the qualitative curvature previously identified in the literature. It did not constrain the structure of the inferential models, which relied on trial-level estimation with appropriate covariate adjustment and robust standard errors, in line with methodological guidance for latency analyses in small samples (Cameron and Miller, 2015; McNeish, 2023).

## Trial-Level Inferential Modeling Framework

### Mean response-time models

The first inferential analyses examined whether stimulation modulated the latency with which participants reported craving across conditions. Response times were log-transformed following the preprocessing procedures described above, which improved distributional symmetry (Baayen and Milin, 2010; Whelan, 2008).

Mean response-latency models were estimated at the trial level, with each trial serving as an individual observation. The fixed effects represented the expected log-latency for each combination of group, time, and stimulus category. The models included trial order and previous-trial latency as covariates to account for within-session temporal dependencies, as established in the preprocessing diagnostics.

Mixed-effects regression was initially selected because trials were nested within participants. Random intercepts and random slopes were tested following standard recommendations for trial-based datasets (Baayen and Milin, 2010; Luke, 2017). However, random-effects models consistently failed to converge or yielded singular variance estimates, indicating that the sample could not support reliable estimation of a hierarchical structure (“MixedLM diagnostics” in Supplementary Script). Consistent with methodological guidance in such contexts (Cameron and Miller, 2015), the final analyses were conducted using ordinary least squares with participant-clustered robust standard errors, ensuring valid inference under within-participant dependence.

To formally quantify the Group × Time interaction, a model-based Difference-in-Differences (DiD) contrast was computed directly from the fitted regression model. Specifically, linear contrasts were applied to the fixed-effect coefficients to estimate the difference between pre–post changes in the lFPC group and those observed in the vertex group, separately for each stimulus category. This approach yields an inferential DiD estimate on the log scale, accompanied by standard errors, confidence intervals, and Wald tests, and corresponds to the primary effect reported in the main manuscript.

In addition, to facilitate interpretation and transparency, a descriptive DiD ratio-of-ratios was derived from model-estimated marginal means. Marginal mean response times were computed on the log scale for each Group × Time × Stimulus condition and subsequently back-transformed to seconds. For each stimulus category, proportional pre–post changes in response time were obtained within each group, and the DiD ratio-of-ratios was calculated by contrasting these changes between the lFPC and vertex groups. This index provides a scale-free descriptive summary of the fitted interaction effect and does not constitute an additional inferential test.

Model diagnostics demonstrated the adequacy of this specification. Residual and Q–Q plots showed approximate normality and homoscedasticity (**Supplementary Figure 3**).

### Curvature models

Curvature analyses quantified whether stimulation modulated the shape of the trial-level relationship between response time and self-reported craving. As in the mean-response models, a mixed-effects specification was initially adopted due to the hierarchical structure of the data (Baayen and Milin, 2010; Luke, 2017). Models included centered linear and quadratic components of trial-level craving deviation (Baayen and Milin, 2010; Germeroth et al., 2015; Mundlak, 1978) and their full interactions with group, time, and stimulus category.

Mixed-effects estimation, however, proved unstable and produced singular random-effect structures, particularly when including polynomial terms and higher-order interactions (“MixedLM diagnostics” in Supplementary Script). Consistent with methodological recommendations for scenarios in which hierarchical models are inestimable, curvature analyses were therefore estimated using ordinary least squares with participant-clustered robust covariance (Cameron and Miller, 2015).

An omnibus model including both stimulus categories was first used to evaluate whether quadratic effects contributed meaningfully to model fit. Follow-up models were then estimated separately for neutral and smoking-related cues, using polynomial terms re-orthogonalized within each stimulus category to obtain interpretable curvature estimates (Baayen and Milin, 2010). Curvature change was defined as the contrast between post- and pre-stimulation quadratic components. Unlike the mean-latency ratio-of-ratios, curvature effects are evaluated as Wald-tested contrasts on the quadratic coefficient (post–pre), within each stimulus category.

In rare cases where stimulus-specific models displayed local rank deficiency or near-collinearity, a simplified, mathematically equivalent parametrization was adopted to stabilize estimation. This alternative specification preserved full three-way interactions for the quadratic term (the component that defines curvature) while reducing the interaction order for the linear term. Importantly, all curvature contrasts were computed identically across parametrizations, and in practice, results from fallback models were numerically consistent with those from the full specification (Baayen and Milin, 2010).

Curvature models additionally included a session-level mean craving term, computed per participant and per time point following a Mundlak-type specification (Mundlak, 1978). This covariate adjusts for stable inter-individual and session-level differences in overall craving intensity, allowing the curvature parameter to isolate within-person fluctuations in craving while avoiding confounding by global changes in subjective craving across pre- and post-intervention sessions (Laurenceau et al., 2025).

Autocorrelation-function analyses confirmed that adjusting for previous-trial latency effectively reduced serial dependence (**Supplementary Figure 4**), supporting the validity of the chosen covariate structure. Multicollinearity checks using variance-inflation factors confirmed negligible correlation among polynomial predictors and temporal covariates (**Supplementary Figure 4**). Robustness analyses employing alternative preprocessing schemes (upper bounds of 10, 15, or 20 seconds; trimming ≥3 SD; reciprocal transformation of response time) yielded consistent results (Supplementary Table 2).

## Clinical association analyses

To investigate whether the behavioral indices derived from the cue-elicited craving task carried clinically meaningful information, we performed exploratory analyses examining their associations with measures of tobacco-use severity. These analyses were conceptually independent from the trial-level models presented in the main text and were designed to determine whether individual differences in response-time dynamics corresponded to broader patterns of chronic craving, nicotine dependence, or smoking behavior.

All clinical associations were based exclusively on pre-intervention behavioral data. As described previously, response-time trials were first restricted to the physiologically plausible range (0.15–20 s) and then log-transformed to stabilize variance and reduce the influence of extreme latencies (Baayen and Milin, 2010; Whelan, 2008). From this prepared dataset, two complementary behavioral indices were computed for each participant and for each stimulus category. The first index reflected the participant’s typical speed when reporting craving and was obtained by calculating the geometric mean of the log-transformed response times. This measure provided a robust estimate of the individual’s overall response-time level at baseline (Baayen and Milin, 2010). The second index characterized how response time varied as a function of moment-to-moment fluctuations in craving intensity. For this purpose, trial-level craving ratings were centered within session, orthogonal polynomial terms were generated, and a quadratic model was fitted to extract the coefficient describing the curvature of the latency–craving function. This coefficient represented the degree of non-linearity in the individual’s response-time profile, indicating whether latencies tended to lengthen at low, intermediate, or high levels of self-reported craving (Baayen and Milin, 2010).

To ensure consistency with the trial-level framework used in the primary models, individual curvature models additionally included trial order and previous-trial latency as nuisance covariates. These terms account for serial dependencies within baseline sessions without altering the interpretation of the quadratic coefficient as a subject-level index of nonlinear response-time dynamics (Baayen and Milin, 2010; Whelan, 2008).

To examine baseline clinical relevance, we computed two subject-level behavioral indices (geometric-mean response time and the curvature coefficient) separately for neutral and smoking cues and correlated them with chronic craving, nicotine dependence, and cigarettes per day using Spearman rank correlations. Analyses were performed separately for each stimulus type. In exploratory partial-correlation models, each association was additionally adjusted for a single confounder (age, years of smoking, depressive and anxious symptoms, impulsivity, or cognitive status), and p-values were corrected for multiple comparisons within stimulus type using the Benjamini–Hochberg false-discovery-rate procedure.

Finally, to explore whether response-time indices could prospectively predict clinical outcomes, we attempted to estimate ANCOVA-type models in which post-intervention clinical scores were regressed on their baseline level together with either the corresponding post-intervention behavioral index or its change from pre to post. These models were estimated separately for each group and stimulus type, employed robust HC3 standard errors, and required a minimum of ten observations per cell to ensure numerical stability and avoid overfitting (McNeish, 2023).

All procedures were fully automated and are documented in Supplementary Script. These analyses were planned as exploratory and were intended to complement, rather than validate, the primary trial-level findings.

## Diagnostic Procedures and Robustness Checks

Complete diagnostic output with residual distributions, Q–Q plots, influence diagnostics, autocorrelation functions, and multicollinearity check for the trial-level models appears in Supplementary Figures 3 and 4. Numerical results from robustness analyses based on alternative thresholds and transformations appear in Supplementary Table 2.

# Supplementary Figures
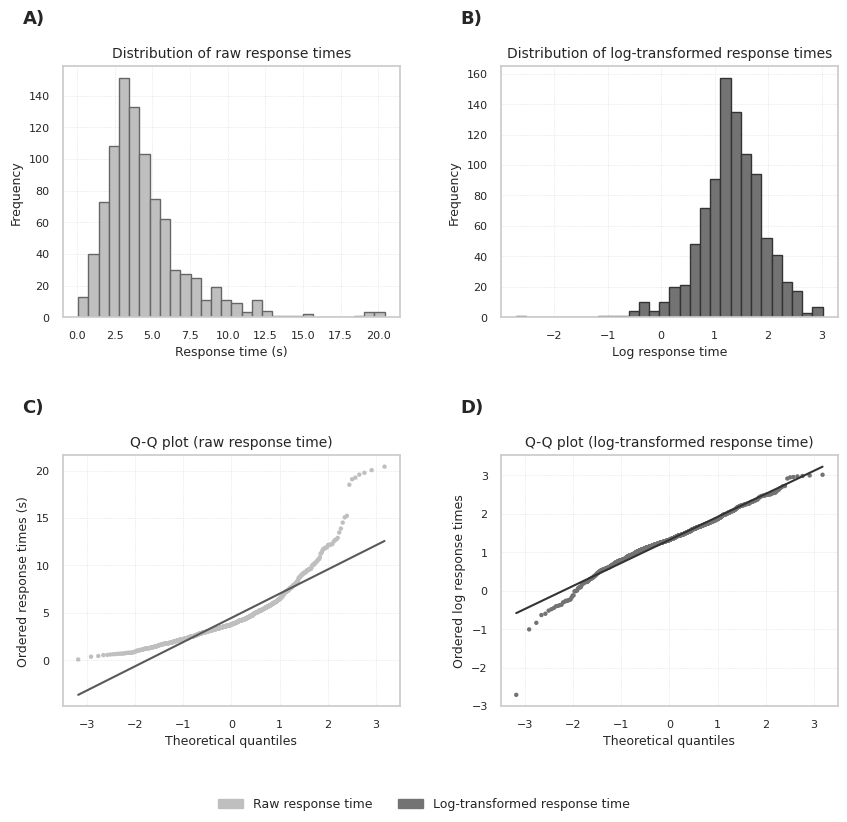


**Supplementary Figure 1**. **Distribution and normality diagnostics for response times.** Panels (A) and (B) show the distributions of raw and log-transformed response times, respectively. Panels (C) and (D) show Q–Q plots comparing ordered values with their corresponding theoretical normal quantiles; points represent observations, and the solid line represents the expected normal distribution.


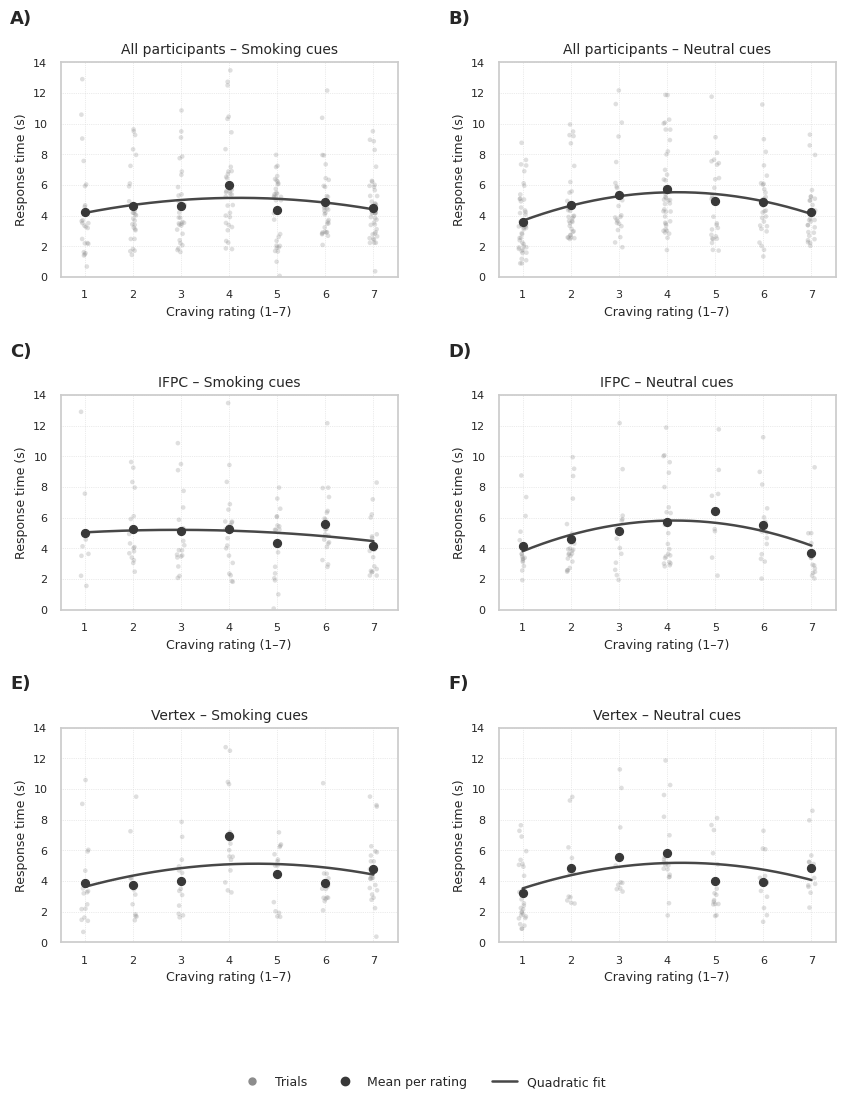


**Supplementary Figure 2. Baseline exploratory response time–craving functions.** Light points represent individual trials; dark points represent mean response time at each craving score; solid lines depict fitted quadratic functions. lFPC: left frontopolar cortex.


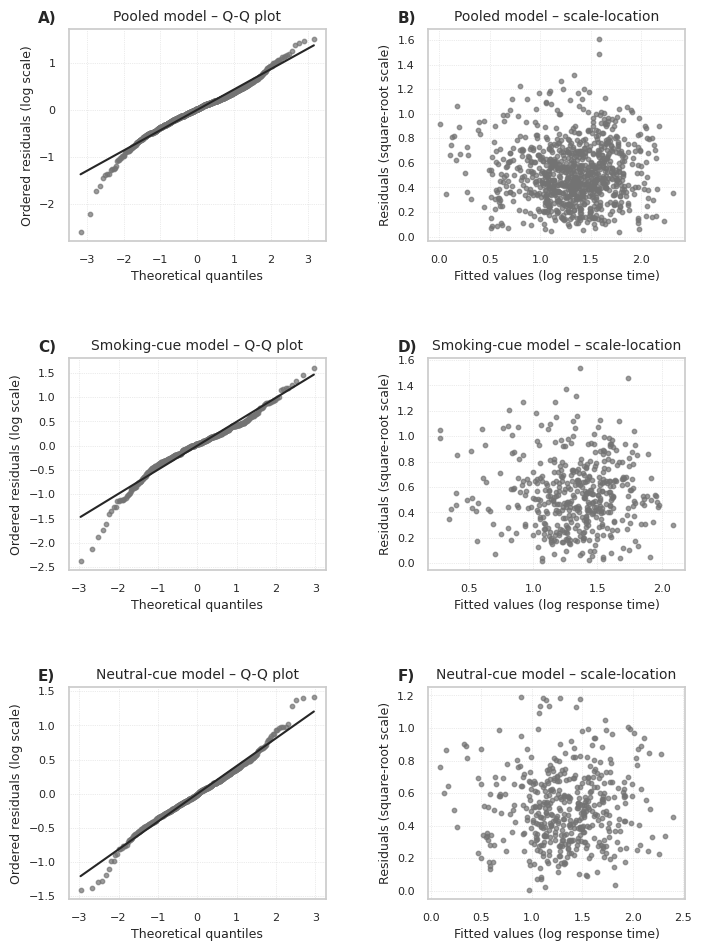


**Supplementary Figure 3. Residual diagnostics for the trial-level response-time model.** Panels (A), (C), and (E) show Q–Q plots of standardized residuals. Points represent ordered residuals, and the solid line represents the theoretical normal distribution. Panels (B), (D), and (F) show scale–location plots of fitted values versus residuals (square-root scale), used to assess the stability of residual variance. All models were estimated with standard errors clustered by participant to account for within-subject dependence.

**
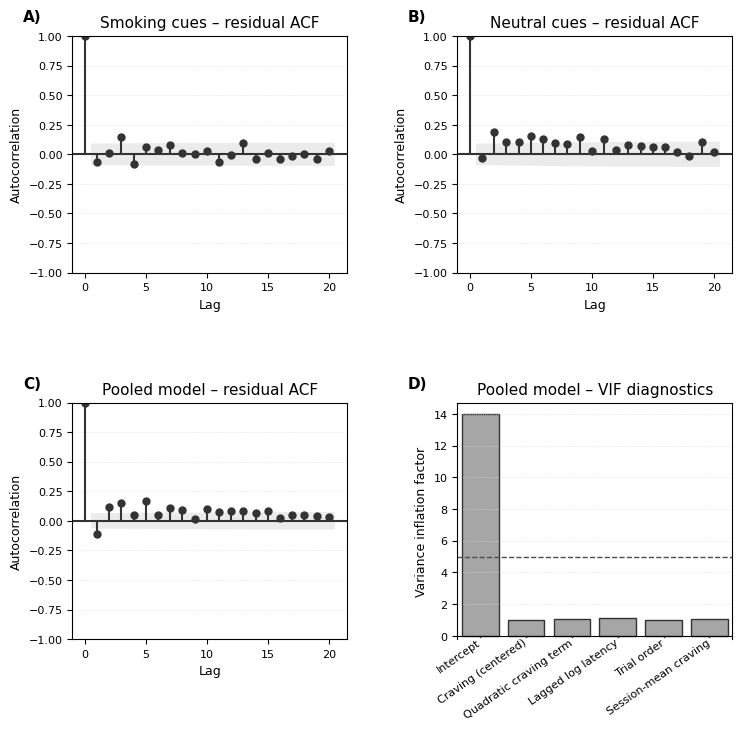
**

**Supplementary Figure 4. Autocorrelation and multicollinearity diagnostics.** Panels (A–C) show autocorrelation-function plots of residuals from the curvature models estimated with participant-clustered standard errors, after adjusting for previous-trial response time. The shaded region represents the 95% confidence interval for autocorrelations under the white-noise assumption. Although some individual lags fall outside the confidence bands, which is expected by chance, no systematic pattern of positive or negative autocorrelation was observed, indicating that short-range serial dependence was effectively minimized. Panel (D) presents variance inflation factors for the continuous predictors included in the curvature specification: centered craving rating (linear term), centered quadratic craving term, lagged log-response latency, trial order, and session-mean craving rating. All predictors showed VIF values below 2, consistent with negligible multicollinearity under the orthogonalized design. The dashed line marks the conventional VIF threshold of 5, above which multicollinearity may become practically relevant. ACF, autocorrelation function; VIF, variance inflation factor.

# Supplementary Tables

**Supplementary Table 1.** Polynomial regressions comparing linear and quadratic baseline fits.

| **Condition** | **Trials (n)** | | **R² adj. (linear)** | **R² adj. (quadratic)** | **β (linear)** | ***p* (linear)** | **β (quadratic)** | ***p (*quadratic)** | **Δ AIC** | **Δ BIC** | **F (ANOVA)** | ***P* (ANOVA)** |
| --- | --- | --- | --- | --- | --- | --- | --- | --- | --- | --- | --- | --- |
| **Pre Neutral** *(pooled)* | | 226 | 0.007 | 0.074 | 1.525 | <0.001 | –0.181 | <0.001 | 14.83 | 11.41 | 17.2387 | <0.001 |
| **Pre Smoking-related** *(pooled)* | | 227 | –0.004 | 0.009 | 0.806 | 0.044 | –0.096 | 0.043 | 2.14 | –1.28 | 4.1232 | 0.043 |
| **Pre lFPC Neutral** | | 118 | –0.003 | 0.071 | 1.674 | 0.001 | –0.202 | 0.001 | 8.13 | 5.36 | 10.3081 | 0.001 |
| **Pre lFPC Smoking-related** | | 119 | –0.001 | –0.006 | 0.259 | 0.668 | –0.044 | 0.527 | –1.59 | –4.37 | 0.4016 | 0.527 |
| **Pre vertex Neutral** | | 108 | 0.010 | 0.057 | 1.320 | 0.006 | –0.154 | 0.014 | 4.19 | 1.51 | 6.1937 | 0.014 |
| **Pre vertex Smoking-related** | | 108 | –0.000 | 0.021 | 1.086 | 0.050 | –0.119 | 0.073 | 1.31 | –1.38 | 3.2637 | 0.073 |

This table summarizes the pre-stimulation relationship between response time and craving intensity using polynomial regression models. For each condition (both pooled across stimulation groups and stratified by lFPC and vertex groups), the table reports adjusted coefficients of determination (R²) for linear and quadratic specifications, standardized linear and quadratic coefficients (β) with their respective p-values, and model-comparison indices (ΔAIC, ΔBIC, and nested-model ANOVA). These analyses quantify the curvature patterns illustrated in Supplementary Figure 2 and provide a numerical characterization of the inverted-U structure observed at baseline. lFPC: left frontopolar cortex; Cz: vertex; ΔAIC: change in Akaike Information Criterion; ΔBIC: change in Bayesian Information Criterion.

**Supplementary Table 2.** Robustness analyses across preprocessing schemes.

| **Scenario** | **Transform** | **Upper bound(s)** | **Trim ≥3SD?** | **n kept** | **% removed** | **β (Neutral)** | ***p* (Neutral)** | **β (Smoking)** | ***p* (Smoking)** |
| --- | --- | --- | --- | --- | --- | --- | --- | --- | --- |
| nofilter_20s | logRT | 20 | No | 917 | 0.33% | –0.0440 | 0.094 | 0.0575 | 0.036 |
| nofilter_20s | invRT | 20 | No | 917 | 0.33% | 0.0144 | 0.331 | –0.0311 | 0.049 |
| trim3sd_20s | logRT | 20 | Yes | 908 | 1.30% | –0.0438 | 0.095 | 0.0595 | 0.029 |
| trim3sd_20s | invRT | 20 | Yes | 908 | 1.30% | 0.0144 | 0.327 | –0.0317 | 0.044 |
| trim3sd_15s | logRT | 15 | Yes | 903 | 1.85% | –0.0469 | 0.087 | 0.0594 | 0.028 |
| trim3sd_15s | invRT | 15 | Yes | 903 | 1.85% | 0.0147 | 0.323 | –0.0317 | 0.043 |
| trim3sd_10s | logRT | 10 | Yes | 873 | 5.11% | –0.0700 | 0.047 | 0.0462 | 0.075 |
| trim3sd_10s | invRT | 10 | Yes | 873 | 5.11% | 0.0181 | 0.358 | –0.0303 | 0.054 |

Estimated quadratic coefficients (β) under alternative preprocessing pipelines, comprising different upper physiological bounds (10–20 s), optional trimming of extreme values (≥3 standard deviations, SD), and alternative response-time transformations (log-transformed [logRT] versus reciprocal [invRT]). Curvature estimates were highly consistent across all preprocessing schemes and cue types (Neutral and Smoking), demonstrating that the observed inverted-U pattern is robust to data-cleaning and transformation choices.
